# Implementation of FRET Spectrometry Using Temporally Resolved Fluorescence: A Feasibility Study

**DOI:** 10.3390/ijms25094706

**Published:** 2024-04-26

**Authors:** Justin Trujillo, Aliyah S. Khan, Dhruba P. Adhikari, Michael R. Stoneman, Jenu V. Chacko, Kevin W. Eliceiri, Valerica Raicu

**Affiliations:** 1 Physics Department, University of Wisconsin-Milwaukee, Milwaukee, WI 53211, USA; trujil24@uwm.edu (J.T.); khan43@uwm.edu (A.S.K.); dpa@uwm.edu (D.P.A.); stonema2@uwm.edu (M.R.S.); 2 Center for Quantitative Cell Imaging, University of Wisconsin-Madison, Madison, WI 53705, USA; jenu.chacko@wisc.edu (J.V.C.); eliceiri@wisc.edu (K.W.E.); 3 Departments of Biomedical Engineering and Medical Physics, University of Wisconsin-Madison, Madison, WI 53705, USA; 4 Morgridge Institute for Research, University of Wisconsin-Madison, Madison, WI 53705, USA

**Keywords:** Förster resonance energy transfer (FRET), FRET spectrometry, spectrally resolved fluorescence, temporally resolved fluorescence, fluorescence lifetime imaging microscopy (FLIM), protein–protein interactions, protein quaternary structure

## Abstract

Förster resonance energy transfer (FRET) spectrometry is a method for determining the quaternary structure of protein oligomers from distributions of FRET efficiencies that are drawn from pixels of fluorescence images of cells expressing the proteins of interest. FRET spectrometry protocols currently rely on obtaining spectrally resolved fluorescence data from intensity-based experiments. Another imaging method, fluorescence lifetime imaging microscopy (FLIM), is a widely used alternative to compute FRET efficiencies for each pixel in an image from the reduction of the fluorescence lifetime of the donors caused by FRET. In FLIM studies of oligomers with different proportions of donors and acceptors, the donor lifetimes may be obtained by fitting the temporally resolved fluorescence decay data with a predetermined number of exponential decay curves. However, this requires knowledge of the number and the relative arrangement of the fluorescent proteins in the sample, which is precisely the goal of FRET spectrometry, thus creating a conundrum that has prevented users of FLIM instruments from performing FRET spectrometry. Here, we describe an attempt to implement FRET spectrometry on temporally resolved fluorescence microscopes by using an integration-based method of computing the FRET efficiency from fluorescence decay curves. This method, which we dubbed *time-integrated FRET* (or tiFRET), was tested on oligomeric fluorescent protein constructs expressed in the cytoplasm of living cells. The present results show that tiFRET is a promising way of implementing FRET spectrometry and suggest potential instrument adjustments for increasing accuracy and resolution in this kind of study.

## 1. Introduction

Quantifying protein–protein interactions is of great interest to biophysicists and other physical and life scientists. Many research strategies and methodologies have been developed and pursued to precisely quantify such interactions, including the nano-scale regime atomic force microscopy (AFM) [1] and luminescence-based bioluminescence resonance energy transfer (BRET) [2]. Another popular method is Förster resonance energy transfer (FRET), which is the non-radiative transfer of energy from an optically excited donor (D) molecule to a nearby acceptor (A) via interactions between their transition dipoles. Since the FRET efficiency, i.e., the fraction of donor excitations transferred to the acceptor, depends on the inverse of the sixth power of the separation distance between D and A [3,4], FRET has been successfully applied to quantifying the homo-oligomerization of proteins tagged with fluorescent molecules [5,6,7,8,9,10]. Of particular interest is computing the distances between D-tagged and A-tagged protomers within oligomers, finding the binding interfaces between protomers, and quantifying the stability of the oligomers.

**Figure 1:**
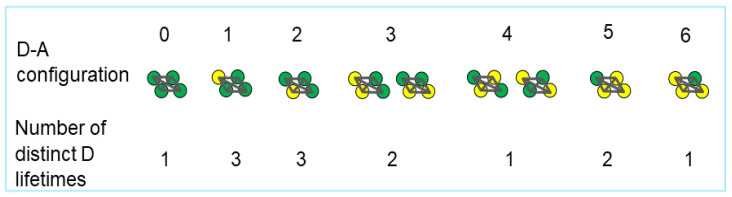
**Illustration of seven possible combinations of donors and acceptors within a parallelogram-shaped tetramer (labelled from 0 to 6).** Each donor (green filled circles) within a parallelogram can transfer energy to one or more acceptors (yellow filled circles) with a rate of transfer that depends on the distance to the acceptors, as described by Förster’s formula [3,4]. The lifetime of the excited state of individual donors, which is equal to the inverse of Förster’s rate of transfer of excitations through FRET, also depends on the distance between said donor and surrounding acceptors. Each configuration can therefore present more than one lifetime; the number of lifetimes is specified under each configuration.

Two broad categories of laser-scanning microscopes are commonly used in FRET experiments: (*i*) intensity-based instruments with spectral resolution and (*ii*) instruments with temporal resolution.

(*i*) Spectrally resolved microscopes collect the emission spectrum of the excited sample and then separate the composite signal into donor and acceptor spectra [11], which have been used for the determination of the quaternary structure of several membrane proteins [5,12,13]. Spectral resolution has been key to the development of *FRET spectrometry* [14,15,16,17], a method that generates distributions of pixel-level FRET efficiency values, as opposed to averages over multiple cells, which are negatively impacted by heterogeneities in the distribution of the molecules of interest within cells [18]. For every quaternary structure, several possible ways exist for placing the donors and acceptors within an oligomeric complex (see Figure 1), and each such oligomer configuration is characterized by an apparent FRET efficiency, *E_app_*, which represents the average FRET efficiency per donor. The *E_app_* value for each D-A configuration depends on the number of donors per oligomer as well as the distance from each donor to each of the acceptors to which the particular donor can transfer energy through FRET. By measuring *E_app_* at the pixel level and computing the frequency of occurrence of pixels with each *E_app_* value, a FRET distribution (or *spectrogram*) is obtained. Such a FRET spectrogram, in which each oligomeric configuration is represented by a peak, constitutes a unique ‘fingerprint’ of an oligomeric structure [16,19]. Detailed geometrical parameters are then derived from the quaternary structure model that best fits the FRET spectrogram [14,20].

(*ii*) The second category of instruments resolves, on a sub-nanosecond timescale, the arrival times of the photons from the excited sample to a detector, from which fluorescence decay histograms are assembled for donors at each image pixel [21,22,23,24,25]. When fitted to one or more exponential decay functions, these histograms provide the excited state lifetime of the donor calculated at the pixel level; this technique is called fluorescence lifetime imaging microscopy (FLIM) [25,26,27]. FLIM has been used to probe a wide range of biologically important interactions including protein localization in living tissues [28], the dynamics of FRET-based biosensors [29], and oligomerization of proteins, such as the B zip domain of the transcription factor CCAAT [30], among many other applications.

If the fluorescently tagged proteins of interest are known to form stable dimers, in FLIM-FRET investigations the FRET efficiency, *E*, is computed from the formula

(1) $$E=1-\frac{\tau^{DA}}{\tau^{D}},$$

where $\tau^{DA}$ is the decay lifetime of the donor in the presence of the acceptor, which is obtained from fitting an exponential function to the fluorescence decay curve of a sample containing the dimers, while $\tau^{D}$ is the lifetime of the donor obtained from fitting the decay curve of a sample containing the donor alone [31].

The kinetic theory of FRET [32,33] predicts that for stable oligomers comprising several donors (see, e.g., Figure 1) the fluorescence decay curve will be composed of a number, *p*, of exponentials, each corresponding to a donor interacting with acceptors located at different distances and having different relative orientations [33]. For example, each of the seven oligomer configurations in Figure 1 is characterized by one to three different lifetimes. In such cases, the following equation needs to be used

(2) $$E=1-\frac{1}{\tau^{D}}\sum_{i=1}^{p}\frac{a_{i}}{\sum a_{i}}\tau_{i}^{Da},$$

where the parameter $\tau^{DA}$ in Equation (1) is replaced by the weighted average of the individual lifetimes of each donor, $\tau_{i}^{Da}$, obtained from a multi-exponential fit, and $a_{i}$ is the amplitude of *i*th exponential in the fit [21,34].

The need to choose an appropriate number of exponential decays to fit to the data leads to the intrinsic requirement that FLIM-FRET experimentalists must *a priori* know the correct number of lifetimes present in the decay to estimate the model and fit the experimental fluorescence decay curve. This creates a vicious cycle, since the number of different donor lifetimes can be inferred only from knowledge of the quaternary (or oligomeric) structure, which is the very information that needs to be extracted from the analysis of the experimental data. The situation becomes even more complicated if the oligomers associate and dissociate into smaller units during the time it takes to acquire the information for a single pixel in an image, or if more than one type of oligomer is present at each pixel, in which case the number of distinct lifetimes that need to be fitted becomes even larger.

To avoid this conundrum and allow for temporally resolved fluorescence measurements to be used in connection with FRET spectrometry, a new method was proposed previously [33], which we aim to experimentally implement and test herein. This method, which we dubbed *time-integrated FRET* (tiFRET), relies on integrating the fluorescence decay curves, instead of fitting a predetermined number of lifetimes to them, which then allows the FRET efficiency to be computed from the area under the fluorescence decay curve using the expression

(3) $$E=1-\frac{1}{\tau^{D}}\frac{\int_{0}^{\infty }p_{D^{*}a}dt}{p_{D^{*}0}},$$

where $p_{D^{*}a}$ is the decay curve of the donor in the presence of acceptors, $p_{D^{*}0}$ is the amplitude of this curve, and $\tau^{D}$, as in Equations (1) and (2), is the excited-state lifetime of the donor in the absence of acceptors, which is extracted from an analysis of fluorescence decay curves obtained from samples containing donors only [33]. Since this expression uses the same fluorescence signal obtained in classical FLIM-FRET measurements, the spatial resolution of the sample and its spectral properties are not affected or misinterpreted by this method of obtaining the FRET efficiency, and so one would still be able to reliably quantify interactions at 10 nm or less, as expected with FRET measurements [12].

**Figure 2:**
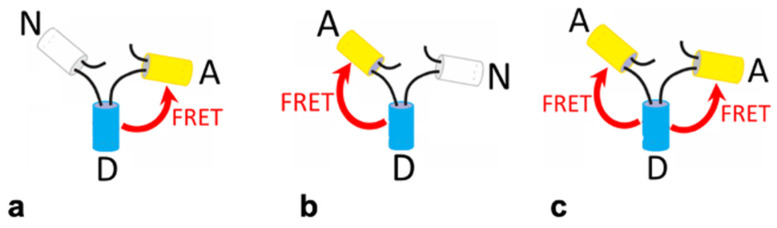
**Illustration of the FRET constructs.** White cylinders represent the non-fluorescent (Amber-N) placeholder protein, cyan cylinders represent the donor protein (Cerulean-D), and the yellow cylinders represent the acceptor protein (Venus-A). The red arrow shows the transfer of energy via FRET from the excited donor molecule to the acceptor. The three trimeric FRET construct arrangements used were (**a**) NDA and (**b**) ADN, each having one donor and one acceptor, and (**c**) ADA, which has one donor and two acceptors.

The results of testing and calibrating the tiFRET method on CHO cells expressing obligate trimeric FRET constructs in the cytoplasm are presented in the following sections. These trimers, originally created at the National Institutes of Health (NIH) by Dr. Stephen Vogel’s laboratory [34], consist of the fluorescent proteins Cerulean (acting as the donor, D) and Venus (acting as the acceptor, A), and two of them also incorporate a non-fluorescent placeholder protein Amber (denoted N). The constructs contain these proteins in the arrangements ADN, NDA, and ADA, as shown in Figure 2.

The constructs ADN and NDA are expected to give similar FRET efficiencies as they have one donor and one acceptor protein. The ADA construct is expected to give a higher FRET efficiency since there are two acceptor proteins and one donor, thereby increasing the likelihood of resonance energy transfer taking place. In addition, the FRET efficiencies of the fluorescent constructs containing only two fluorescent proteins are used to determine the FRET efficiency of the third, via the kinetic theory of FRET [15,32], which allows for additional cross-checking of the results.

Using this biological system as a testbench, we conclude that tiFRET potentially could be used for implementing FRET spectrometry on systems of homo-oligomers with a previously unknown structure, if care is taken to factor in the interpretation of the effects of photobleaching of the fluorescent molecules during measurements. Future refinements in time-resolved fluorescence imaging could also incorporate different sample scanning strategies to reduce the degree of sample photobleaching.

## 2. Results and Discussion

As stated above, the aim of this study is to obtain FRET efficiencies for three covalently formed FRET constructs (ADN, NDA, and ADA) using two different methods—FLIM and tiFRET—in order to validate tiFRET, which could be subsequently used for the determination of FRET efficiencies for transient oligomeric complexes via the method of FRET spectrometry described in the introduction. To do this, we determined pixel-level FRET efficiencies using both the fluorescence lifetime (i.e., FLIM) and time-integrated fluorescence decay (tiFRET) methods for cells expressing separately NDA, ADN, and ADA constructs. For FLIM, we determined FRET efficiencies in two different ways: by fitting the fluorescence decay curves with one and two lifetimes, and then using Equations (1) and (2), respectively, to compute FRET efficiencies for each pixel. Typical pixel-level decay curves and their respective fits with one and two exponentials are shown in Supplementary Figure S1. The decision to use a sum of two exponential decays to fit the data, in addition to a single exponential, seems to be justified by the fact that for some decay curves a single exponential was unable to capture some features in the curves (see below). For tiFRET, the pixel-level FRET efficiency was determined using Equation (3).

The resulting FRET efficiency maps were analyzed using a process of ROI segmentation and meta-histogram construction to extract the final FRET efficiencies for all the samples. This entire protocol, which was introduced and refined on data from spectrally resolved experiments [11,14,35], is summarized in the Materials and Methods section.

**Figure 3:**
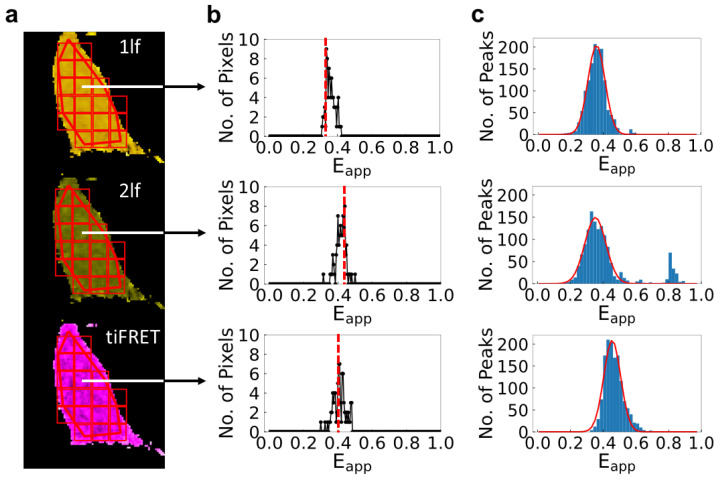
**Illustration of the procedure of image analysis and FRET efficiency meta-histogram construction for cells expressing the FRET construct ADN using three different analysis methods.** (**a**) Regions of interest (ROIs) are drawn onto the intensity images of selected cells. The ROIs are then segmented into squares measuring 10 × 10 pixels using the Moving Square algorithm. The ROIs and segments are then mapped to pixel-level FRET efficiency distribution maps of the cells calculated from the one-lifetime (1lf) fit (top row, yellow cell), two-lifetime (2lf) fit (middle row, green cell), or time-integrated FRET (tiFRET) methods (bottom row, purple cell) (**b**) Histograms of the FRET efficiency values for each segment are constructed and the peak value of each histogram is chosen (indicated by the dashed red line). (**c**) Meta-histograms are constructed from the peak values of the histograms of each segment. For the trimeric constructs, a single FRET efficiency value is expected; therefore, a single Gaussian curve (solid red line) was fitted to the meta-histogram to obtain the FRET efficiency. The R^2^ value for the Gaussian fits to the meta-histograms are as follows: 1lf: 0.99, 2lf: 0.91, tiFRET: 0.97.

A histogram of FRET efficiencies was constructed for each ROI segment, and then a meta-histogram was constructed from the peak values of each ROI segment-level histogram. The meta-histograms were fitted with a single Gaussian function, since each construct was expected to yield one distinct FRET efficiency, and the Gaussian’s mean was taken to represent the most probable FRET efficiency for that sample. The steps of the process and typical results are illustrated in Figure 3.

**Figure 4:**
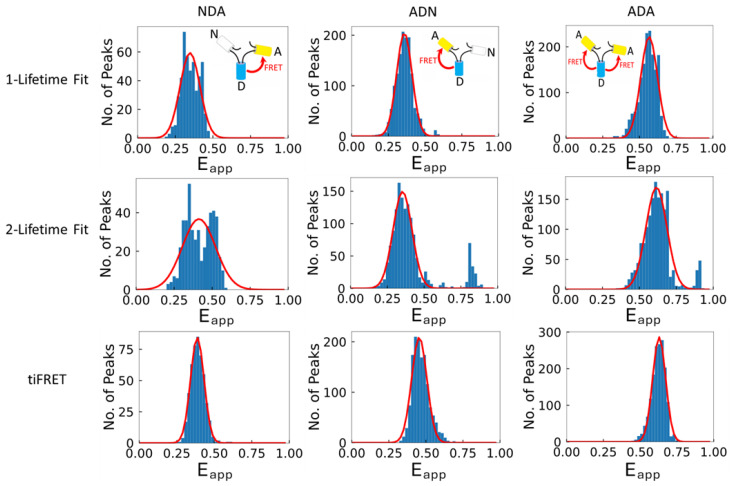
**Typical Gaussian-fitted meta-histograms of the FRET efficiencies for each FRET construct obtained from each of the three calculation methods investigated in this work.** Each meta-histogram was fitted with a single Gaussian function (solid red line) to obtain the mean and standard deviation of the corresponding distribution. The FRET efficiency values ± standard deviation, and the R^2^ values, of the fitted Gaussian shown here from the one-lifetime fit (**top row**) for NDA (**left column**), ADN (**middle column**), and ADA (**right column**) were 0.35 ± 0.07, R^2^ = 0.90; 0.36 ± 0.05, R^2^ = 0.99; and 0.56 ± 0.06, R^2^ = 0.96, respectively. The two-lifetime fit method (**middle row**) gave FRET efficiency values for NDA, ADN, and ADA of 0.41 ± 0.11, R^2^ = 0.76; 0.36 ± 0.06, R^2^ = 0.91; and 0.62 ± 0.07, R^2^ = 0.91, respectively. For the tiFRET method (**bottom row**), the FRET efficiencies for the NDA, ADN, and ADA constructs were 0.39 ± 0.05, R^2^ = 0.99; 0.49 ± 0.05, R^2^ = 0.97; and 0.63 ± 0.04, R^2^ = 0.99, respectively. The one-lifetime fit and to a greater extent the two-lifetime fit methods show deviation from a clear distribution around a single value, while the FRET efficiencies from the tiFRET method do not exhibit this behavior. For these trimeric constructs, a single FRET efficiency value is expected and, therefore, a single Gaussian curve is fitted to the meta-histogram to obtain the FRET efficiency.

Typical meta-histograms obtained for one complete set of measurements employing cells expressing the three different FRET constructs are shown in Figure 4. Note that the two-lifetime fitting process of the fluorescent decay curves was less stable than the one involving a single lifetime, and it resulted in noisier and sometimes bi-modal FRET efficiency distributions. Apart from this, the FLIM-based analyses revealed that it is difficult for the experimenter to decide a priori whether one or two lifetimes should be used, unless knowledge on the structure of the oligomer to be investigated is available. Also noteworthy is that the time-integrated method provided the least noisy FRET efficiency distributions, since no fitting-related noise is introduced through the data analysis process.

In total, four experiments were performed for each of the three FRET constructs, and the results of the individual experiments for each sample type are shown in Supplementary Tables S1–S4. These results were used to compute a weighted average of the FRET efficiency for each construct, via the formula:

(4) $$E=\frac{\Sigma_{k}\frac{1}{\sigma_{k}^{2}}E_{k}}{\Sigma_{k}\frac{1}{\sigma_{k}^{2}}},$$

and standard error of the weighted mean (*SEM*), via

(5) $$SEM={\left(\Sigma_{k}\frac{1}{\sigma_{k}^{2}}\right)}^{-\frac{1}{2}},$$

where $E_{k}$ and $\sigma_{k}$ are the average FRET efficiency and standard deviation, respectively, obtained from the Gaussian fitting of the FRET efficiency meta-histogram obtained for each of the four experiments, indexed by *k*. The combined results of the four experiments are shown in Table 1 along with their corresponding uncertainties. As seen, the results obtained using the tiFRET method gave results that agree with the well-established FLIM-based method, within their respective uncertainties.

Also reassuringly, the FRET efficiencies obtained for each sample behaved as expected from the kinetic theory of FRET [32,33], in that there is a notable increase in FRET efficiency for the ADA construct compared to the NDA and ADN constructs. Previous work on verifying the kinetic theory of FRET using obligate oligomers revealed that the FRET efficiency of the ADA construct can be predicted if the other two FRET efficiencies are known. From the formula for the FRET efficiency for ADA as a function of the other two FRET efficiencies, as well as the corresponding uncertainty given by Patowary et al. [12], we obtain the values 0.49 ± 0.03, 0.58 ± 0.04, and 0.55 ± 0.02 for the FRET efficiencies for the one-lifetime fit, two-lifetime fits, and the integral method, respectively. These predicted values are slightly lower than those obtained from direct measurement of the FRET efficiency of ADA, indicating small systematic errors, likely related to photobleaching, in these measurements (see next paragraph).

To further confirm the accuracy of the results shown in Table 1, we wanted to compare them to the results of a detailed spectrally resolved study performed by some of us [36]. The comparison revealed that the FRET efficiencies determined by FLIM and tiFRET are substantially lower than those obtained from spectrally resolved measurements conducted with a low *excitation power × illumination time* product of 1.2 mW ms (15 mW/pixel), which were 0.53, 0.59, 0.73 (for NDA, ADN, and ADA, respectively). However, they are similar to those obtained for an *excitation power × illumination time* product of 5.0 mW ms (62 mW/pixel). In the separate study, the higher energy deposited in the sample has been shown to produce photobleaching of acceptors via FRET, which suggests that the same may be true of the current setup, although the scanning protocol in the experimental WiscScan system used in this study deposits a similar amount of energy (i.e., about 5.2 mW ms) divided into 60 different doses distributed over the duration of a complete sample scan of 60 s. A more precise comparison between the two methods would require modifying either of the instruments to use exactly the same scanning protocol, which does not seem absolutely necessary for the purpose of the present discussion.

## 3. Materials and Methods

### 3.1. Sample Preparation

Chinese hamster ovarian (CHO) cells were grown in DMEM (Dulbecco’s modified eagle medium, Gibco, Waltham, MA, USA) without sodium pyruvate. The medium was supplemented with 10% fetal bovine serum, 1% penicillin streptomycin, 1% L glutamine and 1% MEM NEAA (non-essential amino acids). Cells were incubated in a humidified environment at 37 °C with 5% CO_2_. Approximately 48 h before imaging, 150,000–200,000 cells were grown on plates coated with poly-D-lysine (Gibco). Cells were transfected with the cytoplasmic expressing trimers containing Cerulean, Venus, and Amber 24 h before imaging using Lipofectamine^TM^ 3000 Transfection Reagent (Invitrogen, Waltham, MA, USA) according to the manufacturer’s protocol. To compute the donor-only decay lifetime, a fourth construct consisting of a Cerulean linked to a non-fluorescent Amber protein was also measured.

### 3.2. Imaging

Transfected cells were imaged in 2 mL of DPBS using a custom-built multiphoton microscope with FLIM capabilities at the Laboratory for Optical and Computational Instrumentation (LOCI, located at the University of Wisconsin-Madison, Madison, WI, USA), as utilized in [37]. The custom-built microscope was equipped with an ultrafast 80 MHz Titanium: Sapphire laser (Chameleon Ultra II, Coherent Inc., Santa Clara, CA, USA). CHO cells were imaged with a 60× oil immersion lens (1.4 NA, Nikon, Melville, NY, USA) and with a two-photon excitation wavelength set at 820 nm; the emission signal was collected using a 457/50 nm bandpass filter (FF01-457/50, Semrock-IDEX, Rochester, NY, USA). The imaging system employed galvanometric scanners to raster-scan the sample with the laser light, a high-gain GaAsP PMT (Hamamatsu, H7422P-40, Hamamatsu City, Japan) to detect the photons emitted by the sample, Becker Hickl TCSPC electronics (SPC-150 board) to determine the photon arrival time, and acquisition software ‘WiscScan’ V7.5 developed at LOCI, University of Wisconsin-Madison, Madison, WI, USA [38] to integrate and control all the components. Acquisition of an entire time-resolved image (256 pixels × 256 pixels) took 60 s to complete and consisted of 60 image scans of about 1 s duration each. During each 1-s scan, each sample voxel (corresponding to an image pixel) was illuminated for 10 µs by the focused laser beam with a power at the sample of <8.7 mW. Thus, each pixel was illuminated for a total of 0.600 ms during the whole 60-s scan series, with average power × illumination time product (or energy) of 5.2 mW ms (or 5.2 µJ). The acquired fluorescence decay curves for each pixel were obtained by adding up the counts from the individual 1-s scans.

### 3.3. Data Reduction

**Figure 5:**
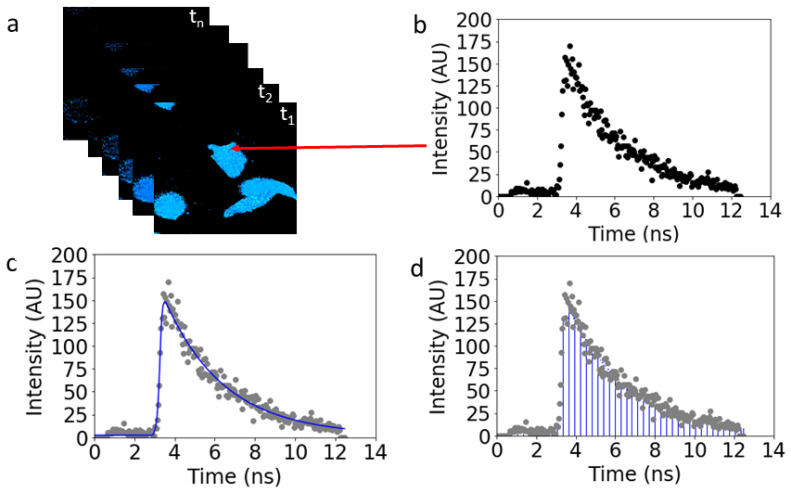
**Comparison of the standard (FLIM) vs. proposed (tiFRET) methods of time-resolved data analysis.** The temporally resolved data (x,y,t) from the Cerulean, Venus, and Amber constructs were collected. (**a**) Image stacks made up of a photon micrograph at each time point were generated. (**b**) Graph of a single pixel in the image stack that corresponds to the measured fluorescence lifetime decay of the fluorescent protein constructs residing in pixel pointed to by red arrow. (**c**) Fluorescence lifetime decay data are fitted using a exponential decay (blue line) to compute the FRET efficiency. (**d**) The integral of the curve allows for the FRET efficiency to be calculated for the donor molecule in the presence of the acceptor using Equation (3).

The collected data, consisting of the sorted photon arrival times for each of the 256 × 256 pixels, were initially analyzed with SPCImage 8.7 (Becker & Hickl GmBH, Berlin, Germany). As illustrated in Figure 5a, an image stack comprised of photon micrographs at each time point was generated. For each pixel in the stack, a time series of the photon count was then plotted vs. time (Figure 5b).

From this point on, two methods of analysis were used. The first method, FLIM, required making assumptions regarding the number of exponential decays exhibited by the data and fit either one or a sum of two such decays to the data experimental curve (Figure 5c). In the second method, tiFRET, which is the one tested in this work, entire fluorescence decay curves were integrated in the case of expression of FRET constructs (Figure 5d). For cells expressing only the donor, the data were fitted with single exponential decays to determine the donor decay lifetime. Lifetime maps obtained from the donor-only lifetimes, $\tau^{D}$, for samples expressing donors only, were needed to compute the pixel-level FRET efficiencies for both approaches. The resultant pixel-level lifetimes and areas under the decay curves, as applicable, were exported to scripts written in Python 3.8.5 (Python Software Foundation) for use in subsequent computation steps to determine the pixel-level FRET efficiency values.

FRET efficiencies were computed as follows. **FLIM:** For samples expressing trimeric Amber, Cerulean, and Venus constructs, one- and two-lifetime fitting analyses were performed with SPCImage separately for each image pixel. Typical results are shown in Supplementary Figure S1. These lifetimes were used to construct one- and two-lifetime FRET maps using Equations (1) or (2) as appropriate, and the lifetime of the donor from samples expressing donors only. **tiFRET:** The FRET efficiency maps were computed, using Equation (3), from the integrals of the pixel-level decay curves and the lifetime of the donor from samples expressing donors only.

### 3.4. Image Segmentation and FRET Meta-Histogram Construction

All FRET efficiency maps were analyzed using a computer program, OptiMiS-DC, developed in-house by us [12,20], which is available at https://sites.uwm.edu/raicu-research-group/software (accessed on 29 March 2024). Regions of interest (ROIs) were drawn around each healthy-looking cell, and these ROIs were further segmented using a moving square algorithm [39]. This algorithm partitions the ROI into square segments, with each segment containing 100 pixels. Each of the four experiments performed for this feasibility study had a varying number of viable cells in each sample, ranging from 11 to 112 cells. However, with the segmentation procedure, the number of segments to analyze for each sample was at least 200 and very often much higher, which provided enough data for this method of analysis.

For the donor-only samples, the ROIs and segments were then mapped to the constructed pixel-level decay lifetime map. A histogram of lifetimes was made for each segment, and the value of the lifetime corresponding to the peak of this histogram was then extracted and stored. A second-level histogram (which is termed the *meta-histogram*) was then assembled from the peak values of the individual histograms for each ROI segment for all the ROIs for a particular sample. To obtain the donor lifetime, $\tau^{D}$, from the sample expressing donors only, a single Gaussian function was fitted to the meta-histogram, and its mean was taken to be $\tau^{D}$. This was then used to compute the various FRET efficiency maps for all other samples using Equations (1)–(3), as appropriate, and data from the cells expressing the oligomeric FRET constructs. FRET efficiency meta-histograms were constructed with the same protocol as the donor-only lifetime maps but instead using the FRET efficiency maps computed in the previous step.

## 4. Conclusions

The ability of the time-integrated FRET method to extract efficiencies of energy transfer from fluorescence images with sub-nanosecond resolution without a need for a priori quaternary structure information has been demonstrated. Compared to classic FLIM, the integration-based calculation of FRET efficiency may reduce the uncertainty inherent in fitting curves as well as in selecting the optimal number of exponential decays to fit the data. This opens the door for current practitioners of temporally resolved fluorescence microscopy to expand their toolkits to include FRET spectrometry, which provides a de nuovo quaternary structure of the protein oligomers. However, one does need to be aware of the general limitation of the method arising from the fact that currently the kinetic theory of FRET, on which this method is based, does not attempt to incorporate multiple decay lifetimes originating from conformational or vibrational substates [40,41], photobleaching effects [42,43], or homo-FRET [44]. Some of these issues may also affect certain intensity-based methods, while the photobleaching issue appears to pose more of a challenge to the temporally resolved measurements, regardless of whether the analysis is in the form of FLIM or tiFRET.

To reduce the impact of donors and acceptors photobleaching on time-resolved FRET measurements, several strategies could be envisioned, including (1) choosing highly photostable molecules as fluorescent markers, (2) implementing different scanning strategies for improving the signal-to-noise ratio without prolonged illumination of the sample, (3) using photon detectors with higher efficiency and thereby reducing the need for high excitation power, and (4) expanding the kinetic theory of FRET to include photobleaching and thereby allowing for incorporation of post-processing corrections. Such goals have been driving ongoing efforts in many laboratories, and numerous pieces of critical work are revealed every day in this area of research.

## Figures and Tables

**Table 1:** Weighted FRET efficiency ± *SEM* computed from all four experiments for each of the three different methods of data analysis for each FRET construct.

Method	Construct		
	NDA	ADN	ADA
1-Lifetime Fit	0.33 ± 0.03	0.32 ± 0.02	0.56 ± 0.02
2-Lifetime Fit	0.43 ± 0.05	0.40 ± 0.04	0.60 ± 0.02
tiFRET	0.36 ± 0.02	0.39 ± 0.02	0.62 ± 0.02

## Data Availability

The data supporting the conclusions of this work will be made available from the authors by request.

## Supplementary Materials

The following supporting information can be downloaded at: https://www.mdpi.com/article/10.3390/ijms25094706/s1.
