# Supplementary material for: Implementation of FRET Spectrometry Using Temporally Resolved Fluorescence: A Feasibility Study
Source: Int J Mol Sci. 2024 Apr 26;25(9):4706. doi: 10.3390/ijms25094706 (PMC11083457; doi:10.3390/ijms25094706)
Supplement: Supplementary file 1 [file ijms-25-04706-s001.zip › ijms-2965737-supplementary.pdf]

Supplementary Materials

Implementation of FRET Spectrometry Using Temporally Resolved Fluorescence: A Feasibility Study

Justin Trujillo [1], Aliyah S. Khan [1], Dhruba P. Adhikari [1], Michael R. Stoneman [1], Jenu V. Chacko [2], Kevin W. Eliceiri [2,3,4] and Valerica Raicu [1,*]

[1] Physics Department, University of Wisconsin-Milwaukee, Milwaukee, WI 53211, USA; trujil24@uwm.edu (J.A.T.); khan43@uwm.edu (A.S.K.); dpa@uwm.edu (D.P.A.); stonema2@uwm.edu (M.R.S.)
[2] Center for Quantitative Cell Imaging, University of Wisconsin-Madison, Madison, WI 53705, USA; jenu.chacko@wisc.edu (J.V.C.); eliceiri@wisc.edu (K.W.E.)
[3] Departments of Biomedical Engineering and Medical Physics, University of Wisconsin-Madison, Madison, WI 53705, USA
[4] Morgridge Institute for Research, University of Wisconsin-Madison, Madison, WI 53705, USA
* Correspondence: vraicu@uwm.edu

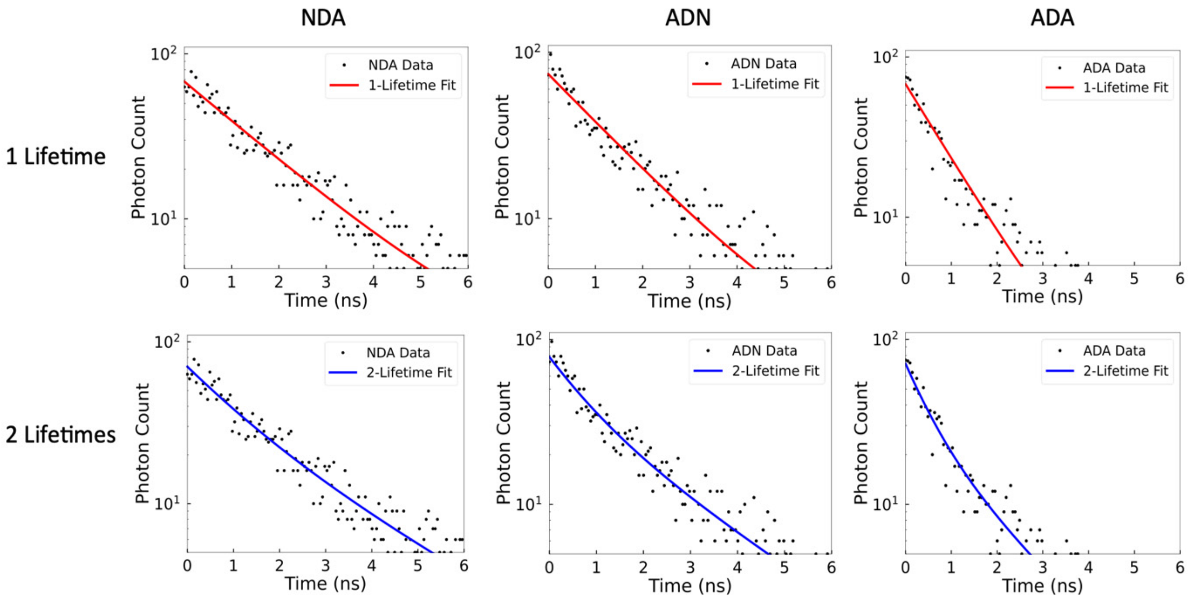

Figure S1. Examples of experimental pixel-level fluorescence decay curves of the fluorescence donors within dimeric and trimeric constructs and their theoretical fit. Semi-log graphs of typical pixel-level fluorescent decay curves for the donor protein for each FRET construct (NDA, ADN, ADA) fitted with one lifetime (top row, red curves) and two lifetimes (bottom row, blue curves). For the curves shown, the values for τ^{DA} from a one-lifetime fit, along with the R^2 values, for NDA, ADN, and ADA were 1.79 ns, $R^2 = 0.95$; 1.48 ns, $R^2 = 0.95$; and 0.92 ns, $R^2 = 0.96$, respectively. The τ^{DA} values obtained from a two-lifetime fit of the decay curves were 1.84 ns, $R^2 = 0.95$; 1.45 ns, $R^2 = 0.96$; and 0.84 ns, $R^2 = 0.97$, respectively.

Table S1. Results of donor-only lifetime and FRET efficiencies (± SD) for Experiment 1. Each experiment was performed following the same cell preparation and experiment protocols.

Method	Donor-Only Lifetime τ^D (ns)	Construct		
		NDA	ADN	ADA
1-Lifetime Fit	2.41 ± 0.14	0.46 ± 0.08	0.46 ± 0.08	0.63 ± 0.05
2-Lifetime Fit	—	0.54 ± 0.08	0.52 ± 0.08	0.66 ± 0.06
tiFRET	—	0.51 ± 0.07	0.49 ± 0.08	0.68 ± 0.04

Table S2. Results of donor-only lifetime and FRET efficiencies (± SD) for Experiment 2. Each experiment was performed following the same cell preparation and experiment protocols.

Method	Donor-Only Lifetime τ^D (ns)	Constructs		
		NDA	ADN	ADA
1-Lifetime Fit	2.33 ± 0.15	0.35 ± 0.07	0.36 ± 0.05	0.56 ± 0.06
2-Lifetime Fit	—	0.41 ± 0.11	0.36 ± 0.06	0.62 ± 0.07
tiFRET	—	0.39 ± 0.05	0.46 ± 0.05	0.63 ± 0.04

Table S3. Results of donor-only lifetime and FRET efficiencies (± SD) for Experiment 3. Each experiment was performed following the same cell preparation and experiment protocols.

Method	Donor-Only Lifetime τ^D (ns)	Constructs		
		NDA	ADN	ADA
1-Lifetime Fit	2.23 ± 0.09	0.21 ± 0.08	0.28 ± 0.03	0.41 ± 0.07
2-Lifetime Fit	—	0.26 ± 0.13	0.36 ± 0.06	0.46 ± 0.09
tiFRET	—	0.26 ± 0.05	0.32 ± 0.04	0.48 ± 0.07

Table S4. Results of donor-only lifetime and FRET efficiencies (± SD) for Experiment 4. Each experiment was performed following the same cell preparation and experiment protocols.

Method	Donor-Only Lifetime τ^D (ns)	Constructs		
		NDA	ADN	ADA
1-Lifetime Fit	2.60 ± 0.28	0.32 ± 0.04	0.36 ± 0.05	0.56 ± 0.03
2-Lifetime Fit	—	0.33 ± 0.12	0.40 ± 0.10	0.60 ± 0.03
tiFRET	—	0.36 ± 0.04	0.40 ± 0.05	0.61 ± 0.02
